# CONUT Score as a Predictor of Mortality Risk in Acute and Chronic Heart Failure: A Meta-Analytic Review

**DOI:** 10.3390/nu17101736

**Published:** 2025-05-20

**Authors:** Diana Andreea Fărcaș, Anda Cerghizan, Raluca Maior, Andreea-Cornelia Mîndrilă, Monica Tarcea

**Affiliations:** 1 Emergency Institute for Cardiovascular Diseases and Transplantation of Târgu Mureș, 540136 Târgu Mureș, Romania; iacobdiana93@gmail.com; 2 Clinic of Internal Medicine II, Emergency Clinical Hospital of Târgu Mureș, 540139 Târgu Mureș, Romania; mtar147@yahoo.com; 3 Department of Clinical and Surgical Disciplines, Faculty of Medicine, G.E. Palade University of Medicine, Pharmacy, Science and Technology from Târgu Mureș, 540142 Târgu Mureș, Romania; 4 Doctoral School, G.E. Palade University of Medicine, Pharmacy, Science and Technology from Târgu Mureș, 540142 Târgu Mureș, Romania; ralumaior@gmail.com; 5 Department of Community Nutrition and Food Safety, Faculty of Medicine, G.E. Palade University of Medicine, Pharmacy, Science and Technology from Târgu Mureș, 540142 Târgu Mureș, Romania; monica.tarcea@umfst.ro

**Keywords:** heart failure, CONUT score, malnutrition, nutritional status, mortality, risk stratification, nutritional screening

## Abstract

Heart failure (HF) is a major global health burden and a leading cause of morbidity and mortality. Nutritional status has emerged as an essential factor influencing outcomes in HF, with the Controlling Nutritional Status (CONUT) score gaining attention as a simple, objective marker derived from serum albumin, total cholesterol, and lymphocyte count. This meta-analysis evaluated the prognostic value of the CONUT score in predicting all-cause mortality in patients with acute and chronic heart failure. A systematic search was conducted in the PubMed, MEDLINE, Google Scholar, and Cochrane Library databases for the past ten years, using combinations of keywords such as “heart failure”, “CONUT score”, “malnutrition”, and “mortality”. Studies were included if they reported hazard ratios (HRs) for all-cause mortality in relation to CONUT score categories in adult HF populations. Eight eligible studies comprising 15,761 patients were included. Pooled analysis showed that higher CONUT scores were significantly associated with increased all-cause mortality (pooled HR = 1.47; 95% CI: 1.30–1.66). Despite substantial heterogeneity (*I*^2^ = 80%), the direction of effect was consistent across studies. The CONUT score is a useful prognostic marker in acute and chronic heart failure patients. Further research should explore the effects of targeted nutritional interventions in high-risk HF patients identified by elevated CONUT scores and efforts to standardize malnutrition cut-offs in clinical practice.

## 1. Introduction

Heart failure (HF) is a complex syndrome characterized by pump dysfunction, usually due to functional or structural changes [1]. The prevalence of heart failure is continuously growing despite the implementation of the four pillars of heart failure treatment: angiotensin-converting enzyme inhibitors (ACE inhibitors) or angiotensin II receptor blockers (ARBs), beta-blockers, aldosterone antagonists, and SGLT-II inhibitors [2]. According to the ESC-HFA Heart Failure Atlas, the prevalence of heart failure in Europe is 17/1000 cases, with some variations between countries: <12/1000 cases in Greece and Spain, and over 30/1000 cases in Lithuania and Germany [3].

This condition is a widespread source of morbidity, mortality, and hospital readmission [2,4]. Patients with heart failure experience progressively worsening functional status and high mortality risk despite progress in treatment methods and the development of healthcare organizations, alongside a multitude of available therapies [1,2]. The most common etiology is ischemic heart disease, but heart failure can also result from valvular pathology, hypertension, or myocarditis. Heart failure, as one of the most common emergencies, presents a high morbidity and mortality rate [4]. To ensure complete management of these patients, we need to acquire information from blood markers, imaging studies, and nutritional status [5]. The latest European Society of Cardiology guidance from 2021 classifies heart failure into three main categories: heart failure with reduced ejection fraction (HFrEF) < 40%, heart failure with mid-range ejection fraction (HFmrEF) 40–49%, and heart failure with preserved ejection fraction (HFpEF) ≥ 50% [6].

The most important serum markers indicating nutritional status are usually collected during every admission; these include proteins (albumin, transtiretin), hematological indicators (haemoglobin, hematocrit, leukocytes, lymphocytes), and measures of glycemic and lipid status (cholesterol, glycated hemoglobin) [7]. Studies over the past few decades have focused on prevention, with dietary patterns being an essential key point. Additionally, more than 45% of heart failure patients may exhibit varying degrees of malnutrition as assessed through the Mini Nutritional Assessment [5]. In heart failure, variations in nutritional status are influenced by several morphopathological changes. Cardiac remodeling due to neurohormonal activation from increased workload and parietal stress, along with fibrosis and systemic inflammation where TNF alpha and interleukin 6 act as intermediaries, serve as primary promoters of catabolic processes that contribute to cachexia. Furthermore, in diabetic patients, endothelial impairments may coexist, exacerbating coronary perfusion issues [8].

Malnutrițion is frequently seen in heart failure patients, in almost 46% of subjects, depending on the studied population or the tool assessment used. Early evaluation and intervention are essential for improving patients’ quality of life [9].

A tool that has been used over the past several years and has proven its value is the Controlling Nutritional Status (CONUT) score, which includes serum albumin, total cholesterol, and total lymphocyte count to assess the patient’s nutritional status [10].

The CONUT score was introduced in 2005 by Ignacio et al. [10] in a study that focused on creating a tool for supervising the nutritional status of hospitalized patients. The purpose was to offer an easy, cheap, and effective method to assess food-related deficiencies that could impact clinical outcomes [11,12]. The CONUT scoring system has undergone extensive validation through numerous studies across a variety of cancer types, such as gastric, colorectal, lung, head and neck, and gynecological cancers. Throughout the late 2010s and early 2020s, several meta-analyses further affirmed the reliability and robustness of the CONUT score as a valuable prognostic tool in cancer prognosis [13]. It was first explored in the context of heart failure in 2017, when researchers began investigating its association with hospitalization risk and mortality among patients with this condition [13,14,15].

In essence, the score integrates protein status (serum albumin), caloric depletion (total cholesterol), and immune status (total lymphocyte count) [15,16]. Nutritional status is an essential component for the management of chronic diseases. The prognostic weight of CONUT scoring has been observed in multiple studies, most usefully in oncologic patients (gastric and intestinal cancer, lung cancer), heart failure, and chronic kidney disease [16,17,18].

Multiple scoring systems have been proposed to assess nutritional status: SGA, Geriatric Nutritional Risk Index (GNRI), Prognostic Nutritional Index (PNI), and the Mini Nutritional Assessment (MNA) [19]. These tools require multiple clinical and anthropometric data points that are not always readily available. In a recent comparative study, the CONUT score was found to have similar capacities to SGA, but only the CONUT score predicted complications [18,19]. In another study involving 30,000 patients, all the scoring systems predicted mortality, but only CONUT was strongly associated [20].

There have been few studies that have confirmed the utility of the CONUT score in cases of heart failure. The biggest of these studies was conducted on 2500 patients, and a high association between both in-hospital mortality and infectious risk was observed, as well as greater value of the CONUT score [11].

Nutritional status is overlooked in usual clinical practice, probably because it cannot be treated quickly without a pathophysiological mechanism on which we can directly act via a specific therapy. Guideline-based nutritional screening as part of standardized treatment is necessary to enhance patients’ prognosis [8,21].

Heart failure guidelines from 2025 highlight malnutrition as a significant factor influencing disease progression and patient prognosis. Malnutrition is commonly linked to higher hospitalization rates and increased mortality, making it crucial to assess the nutritional status of patients with heart failure. Dietary supplements and interventions to correct nutritional deficiencies are recommended as part of a comprehensive treatment approach [4].

Our study presents data supporting recent observations and confirms the CONUT as an important biomarker for predicting mortality.

This study aimed to examine whether the CONUT score, a simple marker of nutritional status, is associated with the risk of all-cause mortality in patients diagnosed with acute or chronic heart failure. We hypothesized that higher CONUT scores would be significantly associated with increased mortality risk in this population, regardless of heart failure subtype.

## 2. Materials and Methods

### 2.1. Eligibility Criteria and Search Strategy

**Figure 1:**
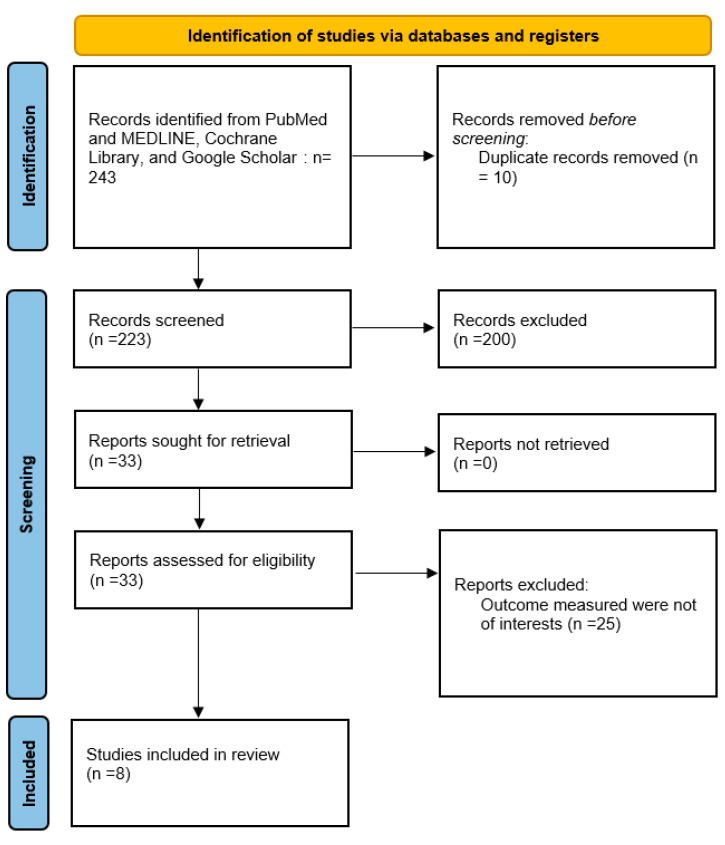
PRISMA diagram [21]. PRISMA flowchart depicting the study selection process. In total, 243 records were identified through electronic databases (PubMed, MEDLINE, Cochrane Library, and Google Scholar). After removal of duplicates, 233 articles were screened, of which 200 were excluded based on title and abstract. Then, 33 articles were assessed for eligibility, with 25 excluded due to irrelevant outcomes or being review articles. Ultimately, eight studies were included in the final qualitative synthesis.

This meta-analysis followed the PRISMA (Preferred Reporting Items for Systematic Reviews and Meta-Analyses) guidelines [21] (Figure 1).

A comprehensive literature search was performed in the electronic databases PubMed and MEDLINE, Cochrane Library, and Google Scholar for studies published between 2015 and 2025 that addressed the prognostic utility of the CONUT score in heart failure patients. Search phrases were as follows: (“CONUT score” OR “Controlling Nutritional Status”) AND (“heart failure” OR “acute heart failure” OR “chronic heart failure”) AND (“mortality” OR “survival” OR “prognosis”).

Eligible studies were observational studies on adult heart failure subjects. We selected only papers that provided hazard ratios (HRs) or alternative outcome measures of the association between CONUT and all-cause mortality. Studies with quantitative outcomes (e.g., hospital readmission or quality of life) were excluded. To account for possible bias, only studies in English were included. Eight studies met the inclusion criteria, involving a total of 15,761 patients.

The inclusion criteria were defined based on the PICO statement. The current study addresses the question: Is the Conut score a prognostic indicator for patients experiencing heart failure regarding overall mortality?

Population: Adults 18 years old with heart failure;

Intervention: High CONUT score;

Comparators: Low CONUT Score;

Outcome: all-cause mortality.

### 2.2. Selection Process

We applied the search terms on the database sites, selecting studies from the previous ten years, and then exported all articles obtained. Two independent authors of our review (F.D.A. and M.T.) screened all titles and abstracts for eligibility. Full-text articles and abstracts with enough information were then assessed independently for inclusion. Any disagreements were resolved through discussion or consultation with a third reviewer. We reviewed the records and reports, choosing the most relevant and appropriate studies. In total, 243 records were identified through electronic databases (PubMed and MEDLINE, Cochrane Library, and Google Scholar). After removal of duplicates, 233 articles were screened, of which 200 were excluded based on title and abstract. Then, the remaining 33 articles were assessed for eligibility, with 25 excluded due to irrelevant outcomes or being review articles. Ultimately, eight studies were included in the final qualitative synthesis.

#### Data Extraction and Quality Assessment

For eligible studies, we extracted the following data into an Excel table: first author, year of publication, study design, country, sample size, age median, CONUT score classification, and reported HRs for all-cause mortality. CONUT scores range from 0 to 12, with higher values indicating poorer nutritional status (Table 1) [22]. To assess the certainty of the included studies, we used the Newcastle–Ottawa Scale (NOS), which evaluates three domains: selection of participants, comparability of cohorts, and outcome assessment. The Newcastle–Ottawa tool classifies studies with ≥8 stars as high quality, 6 to 7 as moderate quality, and ≤5 as low quality, with a maximum of 9 stars [22,23]. All the assessed studies scored ≥ 8 stars. The results are provided in Table 2.

### 2.3. Study Characteristics

Due to expected heterogeneity between studies, we used Comprehensive Meta-Analysis software V4 to prepare a random effects model. We calculated pooled hazard ratios (HRs) for overall mortality, with 95% confidence intervals (CIs). Statistical heterogeneity of the studies was assessed using the *I*^2^ statistic, with *I*^2^ > 50% indicating substantial heterogeneity, and the Cochran’s Q-test, with a *p*-value < 0.10 considered significant. We also conducted subgroup analyses based on heart failure type (acute vs. chronic). Publication bias was assessed using funnel plots and Egger’s test. A sensitivity analysis was conducted to evaluate the robustness of the pooled estimate and to explore whether any single study unduly influenced the overall effect.

The total number of participants in all studies was 15,761, and the central countries were Japan (3), the United States of America (1), Italy (1), China (1), Spain (1), and the United Kingdom (1). The mean age for our studies was 71.83, and most patients were male (62.17%) (Table 2). Study descriptions including the first author, number of participants, country, and effects on the primary outcomes are listed in Table 3. This systematic review and meta-analysis have been registered in PROSPERO (ID: CRD420241043905).

## 3. Results

The eight studies observed mainly focused on measuring the association between the CONUT score and overall mortality in people with acute and chronic heart failure.

### 3.1. Overall Mortality Risk

Pooled hazard ratio (HR) for all-cause mortality associated with elevated CONUT scores was 1.47 (95% CI, 1.30–1.66), signifying about 47% higher mortality in this group. We found significant heterogeneity; the *I*^2^ statistic was 80% among the included studies. The Q-test revealed a value of 35.11 (*p* < 0.01), confirming statistically significant heterogeneity. Table 4 summarizes the individual HRs for each survey, with 95% confidence intervals and statistical weight.

**Figure 2:**
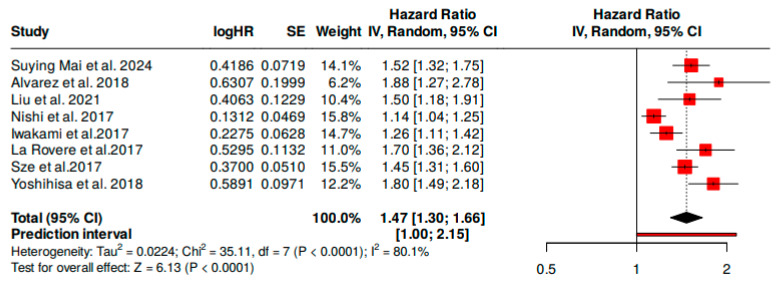
Forest Plot 1 showing pooled HR for all-cause mortality in HF patients with a high CONUT score [9,14,24,25,26,27,28,29]. Hazard ratios (HRs) indicating a statistically increased risk. Study heterogeneity was substantial (*I*^2^ = 80%, *p* < 0.01).

CONUT score was statistically correlated with overall mortality. This meta-analytic assessment showed that the relative risk (HR) was consistently above the threshold HR 1.00, which signifies that the data examined may have been positively associated with the observed outcome. The high heterogeneity in this meta-analysis might be due to different factors such as diversity in the study populations, study design, risk factors, medical comorbidities, measurement protocols, treatment strategies, and methods of analysis (Figure 2). A sensitivity analysis was conducted, and the leave-one-out analysis showed that the exclusion of individual studies did not substantially modify the overall hazard ratio, which ranged from 1.420 to 1.519. All corresponding 95% confidence intervals remained statistically significant, confirming the stability of the findings (Table 5).

Notably, all studies had a consistent direction of association (HR > 1), though the effect size varied. For example, Alvarez et al. reported an HR of 1.88 while Nishi et al. revealed an HR of 1.14, possibly due to patient age, disease severity, or nutritional baseline differences [9,25]. Among the included studies, Nishi et al. [9] and Yoshihisa et al. [29] contributed the highest weights to the pooled analysis (15.3% and 15.1%, respectively). This is likely to have been due to their larger sample sizes and narrower confidence intervals, which increased the statistical precision of their effect estimates. While these studies slightly influenced the overall effect size, sensitivity analysis did not confirm the robustness of the findings when each study was removed individually [9,29].

Using data from the National Health and Nutrition Examination Survey (NHANES) from 1999 to 2018, Mai et al. [24] explored the relationship between nutritional status, assessed via the Controlling Nutritional Status (CONUT) score, and the prevalence of cardiovascular disease (CVD) in individuals diagnosed with chronic obstructive pulmonary disease (COPD). The analysis demonstrated a significant association between higher CONUT scores, indicative of poorer nutritional status, and an elevated risk of cardiovascular mortality. Specifically, each one-point increase in the CONUT score corresponded to a 21% increase in the risk of cardiovascular death (hazard ratio [HR]: 1.52; 95% confidence interval [CI]: 1.32–1.75). These results underscore the prognostic relevance of nutritional screening in patients with COPD, offering critical insights into cardiovascular event risk and long-term outcomes [24].

In a related study, Alvarez et al. [25] reported that patients with impaired nutritional status, as reflected by elevated CONUT scores, were significantly less likely to derive clinical benefit from cardiac resynchronization therapy (CRT). The study found that individuals with higher CONUT scores had a reduced likelihood of treatment success (odds ratio [OR]: 0.57; 95% CI: 0.46–0.71), suggesting that malnutrition may attenuate the therapeutic efficacy of CRT. These findings support the integration of nutritional evaluation into the pre-procedural assessment for CRT candidates [25].

Similarly, Liu et al. [26] identified the CONUT score as an independent prognostic indicator of in-hospital mortality among elderly patients with heart failure. Their results revealed that elevated CONUT scores were significantly associated with increased in-hospital mortality risk (HR: 1.50; 95% CI: 1.18–1.91), with each rise in the CONUT score correlating with an increased risk of all-cause mortality. These data further reinforce the clinical utility of routine nutritional assessment in risk stratification and management of vulnerable cardiac populations [26].

Data from a multicenter registry further also demonstrated that the CONUT score was a valuable predictor of long-term outcomes in patients hospitalized with heart failure, with a hazard ratio (HR) of 1.14 (95% CI: 1.04–1.25) [10]. Iwakami demonstrated CONUT’s superior predictive ability compared to other nutritional markers such as body mass index and C-reactive protein (C-statistic: 0.71). Adding the CONUT score to an existing risk model significantly improved the C-statistic from 0.75 to 0.77 (*p* = 0.02) and enhanced net reclassification (21% for mortality, 27% for survival, 49% overall; *p* < 0.001) [14].

In the context of predicting 12-month mortality in heart failure (HF) patients, Rovere et al. [27] showed that CONUT significantly improved mortality prediction when combined with the MAGGIC score and six-minute walk test (6MWT). These findings suggest that incorporating nutritional assessment into standard HF evaluations enhances risk stratification and should be part of routine clinical practice [27].

Sze et al. [28] studied the prognostic value of frailty and malnutrition indices in patients admitted with heart failure (HF); 265 patients were assessed using three frailty indices and three malnutrition indices (GNRI, CONUT, PNI). The results showed that both frailty and malnutrition were strongly associated with increased mortality, with the worst outcomes observed in patients who were both frail and malnourished. Including the Clinical Frailty Scale (CFS) and Prognostic Nutritional Index (PNI) in the model significantly improved mortality prediction, increasing the c-statistic from 0.68 to 0.84 [28].

Analyzing data from Yoshihisa et al.’s study [29] confirms the association between heart failure and malnutrition, which was closely associated with elevated C-reactive protein levels, tumor necrosis factor-α, adiponectin, B-type natriuretic peptide, and troponin I. Furthermore, both the Prognostic Nutritional Index (PNI) and the Geriatric Nutritional Risk Index (GNRI) outperformed the CONUT score in predicting mortality risk in this population [29].

Overall, the CONUT score has a prognostic value for predicting overall mortality in heart failure patients, but we highlight the necessity of carefully interpreting the results because of variable outcomes. Further research involving larger, well-characterized cohorts and extended follow-up periods is essential to elucidate the prognostic utility of the CONUT score for the clinical management of heart failure.

### 3.2. Subgroup Analyses for the Type of Heart Failure

**Figure 3:**
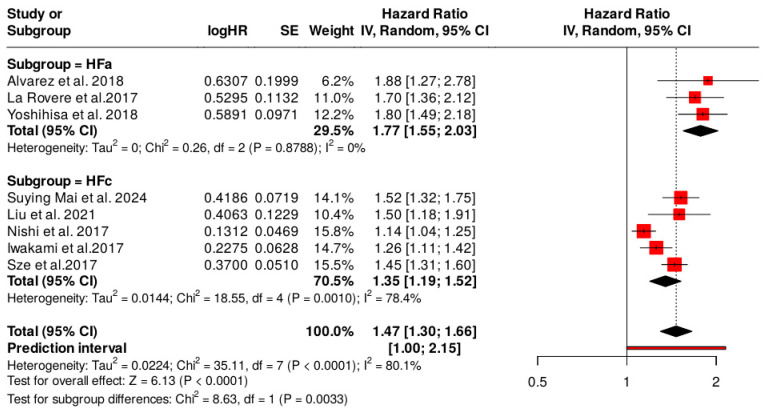
Subgroup analysis: HF_a_ and HF_c_. Hazard ratios with corresponding 95% confidence intervals for studies within the HF_a_ and HF_c_ subgroups, analyzed using a random-effects model [9,14,24,25,26,27,28,29]. The table also includes Z-values, *p*-values, and a forest plot illustrating effect sizes and heterogeneity across studies. Red squares represent the hazard ratio (HR) estimates for individual studies, with square size proportional to study weight. Horizontal red lines denote 95% confidence intervals (CI) for each study. The vertical dashed line indicates the line of no effect (HR = 1). Black diamonds represent the pooled HR with the width corresponding to the 95% CI.

We performed a subgroup analysis to evaluate whether the overall effect varies with heart failure phenotype. Although both subgroups showed significant effects, the magnitude of the impact was noticeably greater in those with acute disease. In the acute heart failure (HF_a_) subgroup, the random-effects analysis indicated a hazard ratio (HR) of 1.775 (95% confidence interval: 1.471–2.142), with a high level of statistical significance (*p* < 0.001). In the chronic heart failure (HF_c_) subgroup, the combined effect was lower, with an HR of 1.347 (95% CI: 1.205–1.506, *p* < 0.001) (Figure 3).

The heterogeneity test showed minimal variability between studies in the HF_a_ subgroup (*I*^2^ = 0%, *p* = 0.883), indicating the homogeneity of the results. In contrast, in the HF_c_ subgroup, heterogeneity was considerable (*I*^2^ = 78.493%, *p* = 0.001), suggesting the existence of essential differences between individual studies.

An interaction test assessed the difference between the effects observed in the two subgroups. The results showed a significant difference between subgroups (Q value = 6.148, df = 1, *p* = 0.013), suggesting that the form of heart failure influenced how patients respond to the intervention (Table 6).

Specifically, the CONUT score has a stronger prognostic impact in patients with HF_a_, showing the importance of stratifying patients according to the type of heart failure when assessing the CONUT score’s effectiveness. The high HR and narrower confidence intervals for the subgroups suggest that we might need to give greater clinical significance to managing patients with heart failure to enhance prognostic accuracy.

### 3.3. Publication Bias

**Figure 4:**
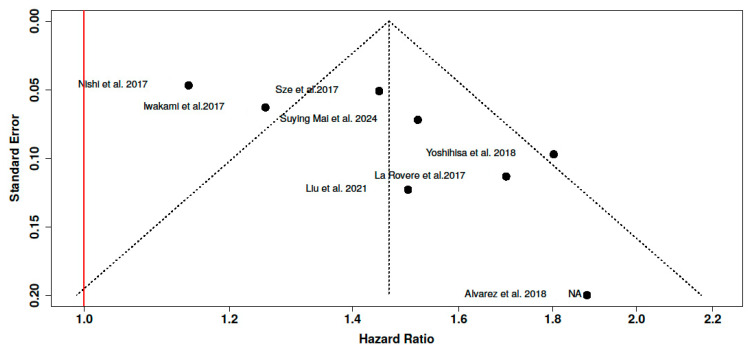
Funnel Plot 2 for publication bias [9,14,24,25,26,27,28,29]. The funnel plot does not indicate a potential publication bias. Dashed lines indicate the 95% confidence limits around the summary effect, helping to assess symmetry, and the Red vertical line represents the null hypothesis (Hazard Ratio = 1), against which publication bias is visually assessed. The horizontal axis represents the hazard ratio (HR), and the vertical axis represents the standard error of the log HR.

No statistically significant publication bias was detected, although the borderline result suggests that some asymmetry cannot be entirely ruled out according to the assessment of funnel plot symmetry and Egger’s test (5% CI: 0.72–6.88, t: 2.419, *p*-value: 0.052). (Figure 4).

## 4. Discussion

Our findings from this meta-analysis indicate essential evidence that the CONUT score is a good prognostic indicator for overall mortality in heart failure patients. A pooled hazard ratio of 1.47 suggests that patients with higher CONUT scores face a 47% greater mortality risk than those with lower scores. These results align with findings from previous studies that have assessed relationships between malnutrition and outcomes in HF patients [30,31]. Bermejo et al. highlight that malnutrition facilitates disease evolution, especially in advanced stages, resulting in a poor prognosis [32].

Each component of the CONUT score is correlated with different stages of heart failure. For example, alteration in lymphocyte counts is a consequence of malnutrition and chronic inflammation and predicts outcomes in coronary artery disease and heart failure [33,34]. Hypoalbuminemia is also caused by both these factors and is an independent predictor of overall mortality [35]. Rauchhaus et al. mentioned in their study that lower serum total cholesterol was associated with worse outcomes for chronic heart failure patients [36].

CONUT scoring has demonstrated its utility in several studies, including studies of chronic kidney disease [37] and oncology [38,39], with strong associations with adverse outcomes. As the paper by Fonarow suggests, the CONUT score reflects the degree of systemic inflammation and nutritional depletion [40].

One notable strength of our study is the inclusion of acute and chronic heart failure patients. In this way, we can observe the CONUT score’s utility in emergency services and in the long-term follow-up of ambulatory patients. Kato et al. clearly indicate that acute heart failure patients with increased CONUT scores in the emergency room had higher mortality and risk of infection [41]. Meanwhile, patients with chronic heart failure, especially with ischemic origin, also had a significant association between malnutrition status and mortality, major cardiac events, re-infarction, or stroke [42]. Probably, chronic inflammation and associated comorbidities like hypertension and diabetes mellitus can explain this relationship [43,44]. Xu et al. mention in their study that in chronic heart failure, the malnutrition status is often impaired by inaccurate body weight measurements due to associated symptoms like edema and proteinuria [45].

Heart failure is a common disease, and treatments focused on reducing biomarkers such as BNP have an essential place in its clinical evaluation. These parameters are routinely obtained during standard clinical assessments. While the CONUT score has demonstrated prognostic significance across numerous studies, B-type natriuretic peptide (BNP) continues to serve as the most established biomarker for evaluating risk and guiding therapy in heart failure, due to its direct association with disease severity and its integral role in patient management. Combined application of CONUT and BNP may provide a more comprehensive assessment of the patient’s clinical status, offering complementary insights into disease progression and long-term outcomes [46].

Our findings are consistent with previous studies that have found the utility of CONUT scoring in other cardiovascular diseases. In acute coronary syndrome and unstable angina, higher CONUT scores predict worse short- and long-term outcomes [41,47]. Bittner et al. proved that CONUT scoring can independently detect patients with a high likelihood of major adverse cardiovascular event (MACE) in stable coronary artery disease [48]. Also, in atrial fibrillation, thromboembolic risk was increased in those with a CONUT score > 4 [49].

CONUT scoring has also proven its value in patients with chronic obstructive pulmonary disease (COPD), being significantly associated with increased prevalence of cardiovascular symptoms and overall mortality [24]. Furthermore, in patients who need cardiac resynchronization therapy (CRT), the CONUT score is an independent factor for hospitalization for acute heart failure and is linked to ventricular remodelling. Monitoring nutritional status post-CRT improves hospitalization and mortality risk [25].

### 4.1. Comparison with Other Prognostic Tools

Other simple nutritional screening tools, such as the Mini Nutritional Assessment (MNA), Prognostic Nutritional Index (PNI), Geriatric Nutritional Risk Index (GNRI), and Subjective Global Assessment (SGA) have also been introduced in clinical practice for the assessment of nutritional state [46]. The GNRI, PNI, and CONUT scores are objective dietary assessment tools that utilize laboratory parameters such as serum albumin, total lymphocyte count, and cholesterol levels. While GNRI emphasizes body weight, PNI reflects immune and nutritional status. In contrast, the Subjective Global Assessment (SGA) relies on clinical judgment, incorporating patient history and physical examination findings [50].

In a recent study involving 500 patients with chronic heart failure, which aimed to compare the predictive value of these tools, CONUT demonstrated the highest sensitivity (80%) for detecting moderate malnutrition. In contrast, MNA-SF and SGA achieved the highest specificity (99%) when evaluated against a composite reference standard [50,51]. On the other hand, Yoshihisa et al. proved that PNI and GNRI were superior in predictive accuracy compared with CONUT [29]. Also, in a recent paper on elderly patients with heart failure with preserved ejection fraction (HFpEF), results showed that PNI had superior predictive ability for mortality compared with CONUT [51].

The prevalence of malnutrition is much greater according to simple screening tools compared with multidimensional tools (diet, weight, lab values, and physical condition), suggesting that the tools measure different aspects of malnutrition. Each may be useful in various situations or for multiple types of malnutrition [52].

### 4.2. Clinical Implications of CONUT in Heart Failure Management

The CONUT score is a simple, cost-effective tool that can assess nutritional status, an essential factor in heart failure. We can better manage other parameters that impact adverse outcomes by lowering systemic inflammation and improving the immune system. Assessment can be conducted in both ambulatory and emergency settings, providing valuable guidance for next steps in treatment [41,43,44].

Consolidating evaluation of nutrition status with CONUT score in HF management provides early detection of at-risk patients, especially the elderly or those with advanced disease, allowing timely nutritional interventions and multidisciplinary support [53].

Repetitive presentation of patients and dietary evaluation can reflect disease progression and the influence of treatment. Nutritional improvement implies not just a dietary approach but a multidisciplinary approach, because in heart failure patients, experiments have altered intestinal absorption, chronic inflammation, and glucose metabolism abnormalities [52,54].

The degree of body wasting is correlated with immune and neurohormonal abnormalities. Anker at all mentions the term “cardiac cachexia” defined as involuntary loss of more than 7.5% of normal weight over a period longer than 6 months, which appears in approximately 16% of patients with chronic heart failure. The metabolic and neurohormonal disturbances associated with malnutrition significantly worsen prognosis in patients with heart failure, underscoring the need for early identification and targeted intervention [55].

The current evidence supports the clinical relevance of the CONUT score as a prognostic indicator in this population. Utilizing routinely measured laboratory markers including serum albumin, total cholesterol, and lymphocyte count, the CONUT score provides a simple, accessible, and cost-efficient means to stratify risk, especially in acute settings where timely decision-making is crucial. It also holds value in long-term outpatient care, where gradual nutritional decline might otherwise go unnoticed [56].

Malnutrition is often exacerbated by systemic inflammation and cardiac cachexia, which both reflect and accelerate the trajectory of heart failure, contributing to reduced functional capacity and poorer clinical outcomes. Early recognition of subclinical malnutrition through tools such as CONUT may prompt timely nutritional support and multidisciplinary interventions, potentially altering the clinical trajectory of these patients. Prior studies have demonstrated that structured nutritional interventions are associated with reductions in rehospitalization and improvements in exercise tolerance and quality of life [11,54]

### 4.3. Integration with Other Prognostic Markers

The prognostic relevance of the CONUT score appears to increase when assessed alongside established cardiac biomarkers. B-type natriuretic peptide (BNP) and *N*-terminal proBNP (NT-proBNP) are well-established indicators of hemodynamic stress and cardiac dysfunction. They are widely used in both heart failure diagnosis and risk stratification [57].

Recent studies suggest that combining nutritional and hemodynamic markers can enhance risk discrimination. For example, Nakashima et al. found that patients presenting with elevated NT-proBNP levels and a high CONUT score exhibited significantly higher all-cause mortality, highlighting the added prognostic value of assessing nutritional status [58]. In a separate investigation, Yamamoto et al. [20] demonstrated that incorporating CONUT alongside BNP improved stratification in patients with heart failure with preserved ejection fraction (HFpEF). In this subgroup, traditional markers often offer limited prognostic clarity.

In addition to the analysis of natriuretic peptides, integrating the CONUT score into multivariable clinical tools, such as the Seattle Heart Failure Model or MAGGIC score, may provide a more comprehensive view of patient risk, particularly in elderly or frail populations. Moreover, there is growing interest in the potential utility of integrating the CONUT score with markers of systemic inflammation, such as C-reactive protein (CRP) and interleukin-6, to more accurately capture the complex relationship between malnutrition, immune activation, and cardiac dysfunction [12,59]. From a clinical standpoint, these combined approaches may offer a refined method for identifying patients who could benefit most from more intensive therapeutic strategies, including targeted nutritional support. While these multidimensional models are theoretically promising, prospective validation is necessary to determine whether such risk-guided interventions can translate into improved clinical outcomes [10,12].

### 4.4. Heterogeinity

The substantial heterogeneity identified in this meta-analysis (*I*^2^ = 80%) reflects notable variability across the eight included studies and warrants careful interpretation. A key source of this heterogeneity lies in the diversity of the patient populations examined. While some studies focused on individuals with acute heart failure [25,27,29], others recruited predominantly chronic heart failure cohorts [9,14,24,26,28], and one included a mixed patient population. This clinical variation is significant, as the prognostic impact of nutritional status may differ according to heart failure subtype and severity.

Further contributing to heterogeneity were differences in baseline characteristics across studies, including age distribution, sex, prevalence of comorbidities such as diabetes, chronic kidney disease, chronic obstructive pulmonary disease (COPD), and variation in left ventricular ejection fraction. These factors are likely to have influenced both nutritional assessment scores and mortality outcomes. Additionally, the duration of follow-up varied widely, from under one year to several years, which may have affected the temporal relationship between malnutrition and mortality.

Methodological differences also played a role. Although all studies employed the CONUT score, there was no uniform definition of malnutrition or consistent categorization of CONUT risk levels. In the study by Alvarez et al., participants were classified into three groups: normal nutritional status (CONUT 0–1), mild malnutrition (CONUT 2–4), and moderate-to-severe malnutrition (CONUT ≥ 5) [25]. Similarly, Yoshihisa et al. adopted a four-tier classification: normal (0–1), mild (2–4), moderate (5–8), and severe malnutrition (9–12), with a commonly used cutoff of 5 to distinguish higher risk [29].

Suying Mai et al. divided their cohort into two categories: normal nutrition (CONUT 0–1) and malnutrition (CONUT ≥ 2) [24], while Sze et al. used a binary classification with a cutoff of >4 to indicate nutritional risk [28]. Nishi et al. used the four-tier classification but applied a statistical cutoff at 5.5 for outcome prediction [9]. In Liu et al., the optimal threshold was determined to be 5.5, based on ROC curve analysis [26].

Iwakami et al. described the CONUT score (range 0–12) as a composite index incorporating serum albumin, total cholesterol, and lymphocyte count, with higher scores indicating worse nutritional status [14]. La Rovere et al. used qualitative categories such as well-nourished, mild/moderate, and severe malnutrition to describe CONUT strata without detailing specific numeric cutoffs [27].

This heterogeneity in CONUT classification schemes and thresholds may contribute to inter-study variability and should be considered when interpreting pooled results.

Nevertheless, the overall trend was uniform: higher CONUT scores were consistently associated with an increased risk of all-cause mortality. Notably, the study by Alvarez et al. reported a relatively high hazard ratio (HR) of 1.88, suggesting a strong link between poor nutritional status and mortality risk [25]. In contrast, Nishi et al. observed a more modest HR of 1.14, indicating that the prognostic utility of the CONUT score may be attenuated in patients with less severe heart failure [9].

Yoshihisa et al. and Iwakami et al. highlighted that patients with higher CONUT scores have increased mortality risk, especially older patients [14,29].

Iwakami et al. and La Rovere et al. also reported minimal sex-based prognostic variance in CONUT scores. The association of CONUT score with increased mortality in heart failure patients was similar in both genders [14,27].

The substantial heterogeneity observed in this meta-analysis (*I*^2^ = 80%) indicates considerable variability across studies and should be considered when interpreting the results. While the direction of the association between higher CONUT scores and increased all-cause mortality was consistent, the magnitude of the effect may differ depending on patient population, study design, follow-up duration, and how malnutrition is defined. Such heterogeneity reduces the precision of the pooled estimate and suggests that the actual effect size may vary in different clinical contexts. Future research should aim to reduce heterogeneity through standardized definitions of nutritional risk and by stratifying analyses based on heart failure phenotype, comorbidities, and treatment setting.

The CONUT score provides some prognostic information, but left ventricular function, comorbidities, and symptomatology should be considered first when evaluating a heart failure patient. The overall consistency of the results supports the conclusion that the CONUT score serves as a valuable prognostic indicator in patients with heart failure. However, the substantial heterogeneity observed across studies underscores the need for standardized definitions of nutritional risk, uniform CONUT score thresholds, and consistent reporting methodologies in future research.

### 4.5. Strengths and Limitations of the Review

Our study reveals robust evidence about the role of nutritional status in acute and chronic heart failure. We demonstrated that the CONUT score is a significant predictor and a key factor in heart failure management, but randomized trials and studies with more available and exact data are needed. Also, the predictive value of different degrees of malnutrition reflected by the CONUT score was not evaluated, because of insufficient data. The absence of data about variables like LVEF, BMI, and functional classification (NYHA) may have contributed to overestimation of the predictive values of CONUT scoring. Another limitation of our study is the restriction to English-language publications, which may have introduced language bias. This may have led to the exclusion of relevant studies published in other languages, potentially affecting the completeness and generalizability of the findings. Our findings are consistent with but also extend beyond those reported in previous meta-analyses evaluating the prognostic value of CONUT scoring in heart failure populations. Another meta-analysis demonstrated a significant association between higher CONUT scores and increased mortality in patients with heart failure. However, that analysis included fewer patients and the study did not include subgroup analyses by heart failure type nor did it assess heterogeneity or publication bias in detail. Also, it included studies published up to early 2020 [11].

Huang et al. reinforced the association between malnutrition and adverse outcomes; they focused on major adverse cardiovascular events (MACEs) and also reported high heterogeneity (*I*^2^ = 81%), which may have affected the precision of their estimates [12].

In contrast, our meta-analysis focused exclusively on hazard ratios (HRs), ensuring methodological consistency. Moreover, we included recently published studies up to 2024, providing an updated evidence base. Importantly, our analysis offers a focused subgroup comparison between acute and chronic heart failure, which has not previously been explored in depth. We also followed a rigorous methodological protocol, including PROSPERO registration, quality assessment using the Newcastle-Ottawa Scale, and evaluation of heterogeneity and potential publication bias.

Taken together, our results provide a more current and detailed synthesis of the prognostic significance of CONUT scoring. Particularly, the current study addresses gaps in the prior literature regarding heart failure subtypes and consistent effect measures.

### 4.6. Directions for Future Research

Future studies should evaluate how nutritional intervention based on the CONUT score can reduce cardiovascular mortality in heart failure and assess its impact on other cardiovascular pathologies. The link between blood markers and heart failure also needs long-term observation.

## 5. Conclusions

According to the positive correlation with functional status, the CONUT score is significantly associated with mortality and can be used to assess nutritional status in acute and chronic heart failure patients. Healthcare providers may use the CONUT score as a tool in a systematic nutritional risk assessment for best management. Future studies must confirm the use of the CONUT score in clinical settings and the benefits of targeted intervention to improve nutritional status and mortality in patients with acute and chronic heart failure. This meta-analysis showed that the CONUT score is independently associated with overall mortality in heart failure patients. Although there is heterogeneity among studies, the pooled results indicate that patients with higher CONUT scores are at greater mortality risk. Due to its simplicity and availability, CONUT scoring can be an important prognostic marker for clinicians to predict patient outcomes and develop management strategies for patients with heart failure. Prospective investigations are warranted to standardize the routine use of CONUT scoring in clinical practice and to evaluate its role in the context of other prognostic markers.

## Figures and Tables

**Table 1:** CONUT scoring system [23].

Parameters	Normal	Mild	Moderate	Severe
Serum albumin (g/mL)	≥3.5	3.0–3.4	2.5–2.9	<2.50
Score	0	2	4	6
Total lymphocyte count	≥1600	1200–1599	800–1199	<800
Score	0	1	2	3
Total cholesterol	≥180	140–179	100–139	<100
Score	0	1	2	3
Total score	0–1	2–4	5–8	9–12
Dysnutritional states	Normal	Mild	Moderate	Severe

CONUT, controlling nutritional status.

**Table 2:** Study characteristics based on sex and age.

Study	Mean Age (Years)	Male (%)	Female (%)
S1. Suying Mai et al. * [24]	NA	NA	NA
S2. Alvarez et al. [25]	66 ± 10	77	23
S3. Liu et al. [26]	74.8 ± 7.0	50.2	49.8
S4. Nishi et al. [9]	2 ± 12	60	40
S5. Iwakami et al. [14]	78	55	45
S6. La Rovere et al. [27]	67 ± 11	70	30
S7. Sze et al. [28]	76 ± 11	60	40
S8. Yoshihisa et al. [29]	68 ± 13	65	35

* NA = Not available. Age and gender distribution for patients with heart failure in the study by Suying Mai et al. [24] were not reported in the original publication. Data represent mean ± standard deviation, where applicable.

**Table 3:** Characteristics of studies evaluated.

	Study	Author/Year	Type of Study	Country	Quality Assessment Results (Newcastle–Ottawa *)	Sample Size	Effects on the Main Outcomes	Reference
1.	Controlling nutritional status score in the prediction of cardiovascular disease prevalence, all-cause and cardiovascular mortality in chronic obstructive pulmonary disease population: NHANES 1999–2018	Suying Mai/2024	Cohort study	USA	NOS = 9/9	N = 309 people ≥ 35 years of age with COPD and HF from NHANES 1999–2018	A count score > 2 is associated with a high prevalence of cardiovascular disease and overall mortality risk, and studies suggest that it is a good nutritional tool for patients’ risk stratification.	[24]
2.	Prognostic value of nutrition status in the response of cardiac resynchronization therapy.	Alvarez/2018	Retrospective Observational Study	Spain	NOS = 8/9	N = 302	Results showed that those with moderate-severe malnutrition had the highest risk of acute heart failure hospitalization and mortality risk, as well as an association with ventricular remodelling.	[25]
3.	Controlling Nutritional Status Score as a Predictive Marker of In-hospital Mortality in Older Adult Patients	Cheng Liu/2021	Retrospective Cohort Study	China	NOS = 8/9	N = 11,795	CONUT ≥ 6 was associated with a high prevalence of long-term adverse outcomes, proving that it is an independent predictor of mortality, especially among the elderly.	[26]
4.	Nutritional screening based on the controlling nutritional status (CONUT) score at the time of admission is useful for long-term prognostic prediction in patients with heart failure requiring hospitalization	Isao Nishi/2017	Retrospective Observational Study	Japan	NOS = 9/9	N = 482	Analyses revealed that a per-point increase in the CONUT score was associated with an increased risk of all-cause death	[9]
5.	Prognostic value of malnutrition assessed by Controlling Nutritional Status score for long-term mortality in patients with acute heart failure	Naotsugu Iwakami/2017	Retrospective Observational Study	Japan	NOS = 8/9	N = 635	Higher CONUT score at admission was significantly associated with increased long-term mortality. The CONUT score also improved the predictive accuracy of existing risk models.	[14]
6.	Additional predictive value of nutritional status in the prognostic assessment of heart failure patients	M.T. La Rovere/2017	Prospective Observational Study	Italy	NOS = 8/9	N = 466	The results showed that a higher CONUT score was associated with increased mortality over a 12-month period, demonstrating the importance of nutritional evaluation in risk stratification for patients with heart failure.	[27]
7.	Prognostic value of simple frailty and malnutrition screening tools in patients with acute heart failure due to left ventricular systolic dysfunction	S.Sze/2017	Prospective Observational study	United Kingdom	NOS = 9/9	N = 265	The findings indicated that frailty and malnutrition are strongly associated with adverse outcomes, improving mortality prediction.	[28]
8.	Impact of nutritional indices on mortality in patients with heart failure	Akiomi Yoshihisa/2018	Retrospective cohort study	Japan	NOS = 8/9	N = 1307	The results indicated that malnutrition was associated with increased all-cause mortality, with PNI and GNRI demonstrating superior predictive accuracy compared to CONUT.	[29]

* NOS: Newcastle–Ottawa Scale, a tool that classifies studies from a maximum of 9 stars, “≥8 stars, high quality”, “6 to 7, moderate quality”, to “≤5 stars, low quality”.

**Table 4:** The individual hazard ratios and weights for each study.

Study	HR * (95% CI *)	Weight (%)
Suying Mai et al., 2024 [24]	1.52 [1.32–1.75]	11.1%
Alvarez et al., 2018 [25]	1.88 [1.27–2.78]	7.3%
Liu et al., 2021 [26]	1.50 [1.18–1.91]	12.4%
Nishi et al., 2017 [9]	1.14 [1.04–1.25]	15.3%
Iwakami et al., 2017 [14]	1.26 [1.11–1.42]	14.5%
La Rovere et al., 2017 [27]	1.70 [1.36–2.12]	11.7%
Sze et al., 2017 [28]	1.45 [1.31–1.60]	12.6%
Yoshihisa et al., 2018 [29]	1.80 [1.49–2.18]	15.1%

* HR = hazard ratio; CI = confidence interval. Studies are weighted according to their contribution to the meta-analysis.

**Table 5:** Sensitivity Analysis Test.

Study Name	Subgroup Within Study	Point	Lower Limit	Upper Limit	Z-Value	*p*-Value	Hazard Ratio 95%CI with Study Removed		
							0.01	1.00	10.00
Suying Mai et al., 2024 [24]	HF_c_	1.462	1.273	1.679	5.384	0.000		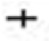	
Alvarez et al., 2018 [25]	HF_a_	1.442	1.274	1.633	5.772	0.000		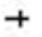	
Liu et al., 2021 [26]	HF_c_	1.465	1.283	1.674	5.633	0.000		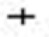	
Nishi et al., 2017 [9]	HF_c_	1.519	1.376	1.676	8.283	0.000		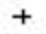	
Iwakami et al., 2017 [14]	HF_c_	1.511	1.310	1.743	5.676	0.000		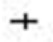	
La Rovere et al., 2017 [27]	HF_a_	1.439	1.267	1.635	5.591	0.000		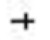	
Sze et al., 2017 [28]	HF_c_	1.479	1.274	1.717	5.134	0.000		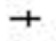	
Yoshihisa et al., 2018 [29]	HF_a_	1.420	1.259	1.601	5.723	0.000		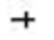	
Random		1.467	1.298	1.657	6.152	0.000		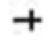	
Pred Int		1.467	0.987	2.180	0.000	0.000			

Leave-one-out sensitivity analysis of the association between CONUT score and all-cause mortality in patients with heart failure. Each row shows the pooled hazard ratio (HR) with 95% confidence interval (CI) when the respective study was removed from the meta-analysis. The consistency of the results across all iterations indicates that no single study significantly influenced the overall estimate.

**Table 6:** Test interaction between HF_a_ and HF_c_.

Groups	Effect Size and 95% Interval				Test of Null (2-Tail)		Prediction Interval		Between-Study		Other Heterogeneity Statistics			
Group	Number of Studies	Point Estimate	95% CI (Lower)	95% CI (Upper)	Z-Value	*p*-Value	Lower	Upper	Tau	TauSq	Q-Value	d*f* (Q)	Q *p*-Value	*I*^2^ (%)
Fixed effect analysis														
HF_a_	3	1.771	1.547	2.028	8.267	0.000					0.248	2	0.883	0.0
HF_c_	5	1.314	1.245	1.386	10.027	0.000					18.598	4	0.001	78.493
Total within											18.846	6	0.004	
Total between											16.178	1	0.000	
Overall	8	1.367	1.301	1.437	12.358	0.000					35.024	7	0.000	80.014
Random effects analysis														
HF_a_	3	1.775	1.471	2.142	5.994	0.000	1.251	2.520	0.106	0.011				
HF_c_	5	1.347	1.205	1.506	5.240	0.000	1.003	1.809	0.106	0.011				
Total between											6.148	1	0.013	
Overall	8	1.530	1.168	2.003	3.089	0.002	0.985	2.127	0.149	0.022				

Results of fixed-effect and random-effects meta-analyses, showing effect sizes, confidence intervals, Z- and *p*-values, prediction intervals, and heterogeneity statistics for HF_a_ and HF_c_ subgroups, including between-study variance (Tau, Tau^2^), Q-tests, degrees of freedom, and *I*^2^ values to assess heterogeneity within and between subgroups.

## Data Availability

All data are contained within the review.

## Abbreviations

The following abbreviations are used in this manuscript:

HF	Heart failure
ACE	Angiotensin-converting enzyme
CRT	Cardiac resynchronization therapy
6MWT	6-min walk test
CFS	Clinical frailty scale
BNP	B-type natriuretic peptide (a marker of heart failure)
LVEF	Left ventricular ejection fraction
BMI	Body mass index
EF	Ejection fraction
CRP	C-reactive protein
MAGGIC	Meta-Analysis Global Group in Chronic Heart Failure
CONUT	Controlling Nutritional Status
SGA	Subjective Global Assessment
GNRI	Geriatric Nutritional Risk Index
PNI	Prognostic Nutritional Index
MNA	Mini Nutritional Assessment
NYHA	New York Heart Association
CVD	Cardiovascular disease
COPD	Chronic obstructive pulmonary disease
ARB	Angiotensin receptor blocker
SGLT	Sodium–glucose cotransporter
ESC	European Society of Cardiology
HFA	Heart Failure Association
TNF	Tumor necrosis factor
PRISMA	Preferred Reporting Items for Systematic Reviews and Meta-Analyses
PICO	Patient, Intervention, Comparison, Outcome (framework for clinical questions)
HR	Hazard ratio
CI	Confidence interval
NOS	Newcastle–Ottawa Scale
OR	Odds ratio
NHANES	National Health and Nutrition Examination Survey
